# Quality of Antenatal care services in eastern Uganda: implications for interventions

**Published:** 2012-10-09

**Authors:** Moses Tetui, Elizabeth Kiracho Ekirapa, John Bua, Aloysius Mutebi, Raymond Tweheyo, Peter Waiswa

**Affiliations:** 1 Department of Health Policy, Planning and Management, Makerere University School of Public Health, P.O.Box 7072, Kampala, Uganda

**Keywords:** Antenatal care, process quality, patient satisfaction, capacity

## Abstract

**Introduction:**

More efforts need to be directed to improving the quality of maternal health in developing countries if we are to keep on track with meeting the fifth millennium development goal. The World Health Organization says developing countries account for over 90% of maternal deaths of which three fifths occur in Sub-Saharan African countries like Uganda. Abortion, obstetric complications such as hemorrhage, dystocia, eclampsia, and sepsis are major causes of maternal deaths here. Good quality Antenatal Care (ANC) provides opportunity to detect and respond to risky maternal conditions. This study assessed quality of ANC services in eastern Uganda with a goal of benchmarking implications for interventions.

**Methods:**

Data was collected from 15 health facilities in Eastern Uganda to establish capacity of delivering ANC services. Observation checklists were used to assess structural components and completeness of the ANC consultation process among 291 women attending it. Lastly, structured exit-interviews were conducted to assess satisfaction of patients. Data analysis was done in STATA Version 10.

**Results:**

There was an overall staffing gap of over 40%, while infection control facilities, drugs and supplies were inadequate. However, there was good existence of physical infrastructure and diagnostic equipment for ANC services. It was observed that counseling for risk factors and birth preparedness was poorly done; in addition essential tests were not done for the majority of clients.

**Conclusion:**

To improve the quality of ANC, interventions need to improve staffing, infection control facilities and drug-supplies. In addition to better counseling for risk factor-recognition and birth preparedness.

## Introduction

The fifth millennium development goal aims at reducing maternal mortality by 75% by the year 2015 [1]. According to the WHO, there was an estimated 358,000 maternal deaths globally in 2008. Developing countries accounted for 99% of these deaths of which three fifths occurred in Sub-Saharan Africa where Uganda lies [2]. Abortion, obstetric complications such as hemorrhage, dystocia, eclampsia, sepsis and infections such as tuberculosis and HIV are the major causes of maternal deaths in developing countries [3]. Although antenatal care (ANC) is not in itself very effective in reducing maternal mortality, it provides an entry for interventions which give health workers the opportunity to detect these risky conditions and therefore refer them for early management leading to better maternal outcomes [4].

ANC involves screening for health and socioeconomic conditions likely to increase the possibility of specific adverse pregnancy outcomes, providing therapeutic interventions known to be effective and educating pregnant women about planning for safe birth, emergencies during pregnancy and how to deal with them [5]. ANC is therefore relevant for the improvement of maternal health as it enables the monitoring of the health of the mother and anticipation of any difficulties during pregnancy, labor and birth [6]. Some studies have estimated that ANC alone can reduce maternal mortality by 20% [7] given good quality and regular attendance. In addition ANC attendance during pregnancy has been shown to have a positive impact on the use of postnatal healthcare services, which also play a key role in detecting risky conditions after child birth consequently leading to better maternal health outcomes [8].

WHO evidence shows that four ANC visits are sufficient for uncomplicated pregnancies and more are necessary only in cases of complications[9]. The WHO, therefore recommends four visits, however in developing countries, many women do not attend all the four visits [10–12]. This has been attributed to poor accessibility, inability to afford the costs of seeking care, cultural barriers and lack of knowledge or illiteracy [13–15].

In Uganda the ANC services are characterized by poor attendance, poor counseling services and poor client-provider relations, with the quality being worse in rural areas [16]. The quality of ANC is critical in enabling women and health workers identify risks and danger signs during pregnancy which should lead to appropriate action[17]. Whether or not women can identify danger signs during pregnancy and act appropriately depends on quality aspects such as the depth of the information and counseling given during an ANC visit [18].

A typical ANC visit in Uganda should involve history taking, clinical examination, running of essential tests including HIV, counseling on risk factors, danger signs and how to handle them, counseling on birth preparation, administration of tetanus toxoid vaccine and other essential supplements [19]. Provision of quality ANC service requires the presence of relevant Infrastructure, adequate trained health workers, infection control facilities, diagnostic equipment, supplies and essential drugs. Furthermore, the ANC process requires the use of guidelines that health providers should follow while offering care to ensure prevention, diagnosis and treatment of complications [20].

This study assessed the quality of ANC services by looking at the health facilities capacity to deliver ANC services, the completeness of the ANC consultation process and patient satisfaction with ANC services offered. It was done by the Future Health Systems consortium as part of the formative work for an intervention that aims at improving access to maternal health services using vouchers. The knowledge gained by this study can be used in the design of interventions aimed at improving the quality of ANC and other maternal health services especially in rural areas of Uganda.

## Methods

### Study design and setting

This was a cross sectional study that was undertaken in two rural districts of eastern Uganda, Kamuli and Pallisa. Kamuli district has a population of 712,079 people with women of reproductive age (15-49) making 20.4% of the population. The district has eight Government health centre IIs, seventeen health centre IIIs and five health centre IVs. More so, it has twenty-three private/NGO dispensaries, thirteen clinics and a hospital. There is also one Mission Hospital. Pallisa district on the other hand has a population size of 522,254 people with women of reproductive age similarly making 20.4% of the population. The district has four Government health centre IIs, twenty-two health centre IIIs, three health centre IVs with 1 hospital. It has six private/NGO dispensaries, twenty-two clinics and six health centre IIIs. The major form of livelihood in both districts is subsistence farming.

### Study population, sampling and data collection

Two health sub districts in each of the districts were randomly selected from a pool of four health sub districts. To assess the capacity of the health facilities to offer ANC services, a total of 15 health facilities were randomly sampled from the health facilities that offer delivery care in the two districts. All the patients that attended ANC during the days of data collection and consented to be part of the study were observed (299) and 291 were interviewed to assess the process quality of the ANC services provided and to establish patient satisfaction with the ANC service respectively.

### Data Collection

Structured interviews with facility managers were carried out to determine the staffing levels and patients’ satisfaction levels with the ANC services provided. For availability of human resources, we established the existing staffing levels for medical doctors, nursing officers, clinical officers, midwives, comprehensive nurses, enrolled nurses and nursing assistants. Observation checklists were used to determine the process quality of the ANC service and to assess the presence of structural elements such as; infection control facilities, physical infrastructure, diagnostic equipment and essential drugs.

Data on the existence of piped running water, other running water, water in a bucket or basin, hand washing soap, disposable hand drying towels, waste receptacle bins with lids and plastic liners, sharps containers, disposable latex gloves, already mixed disinfection solution and unmixed disinfectant was collected to establish the existence of infection control facilities.

For physical infrastructure availability, data was collected on the existence of a waiting area, an examination table, and private rooms for auditory and visual privacy. The diagnostic equipment assessed included; a spotlight, blood pressure apparatus, stethoscope, fetoscope, adult weighing scale and a vaginal speculum whereas the essential drugs included; tetanus toxoid, fansidar, iron and mebendazole.

To assess the process quality of antenatal care offered, it was observed if a client was examined, their history taken, essential tests carried out, and if they were counseled on risk factors recognition and birth preparedness. It was also observed if they were offered treatment and given explanations about the treatment.

Under history taking, it was observed if the clients’ age, current medications, date of last menstrual period and number of prior pregnancies was obtained. In addition it was observed if the client was asked about signs of bleeding, fever, headache or blurred vision, swollen hands and legs, tiredness or breathlessness and baby movement. Secondly we observed the clients’ examination, which involved, taking the client’s age, blood pressure, and palpation of client’s abdomen for foetal lie presentation and fundal height, foetal heartbeat and examination of client’s breasts. Finally, we performed the the following test: the blood test for anemia, syphilis, blood group test, Rhesus factor, urine test for protein and glucose and HIV test. Under counseling for risk factors, it was observed whether or not a client was counseled about, vaginal bleeding, fever, swollen hands and legs, headache, blurred vision, excessive tiredness and breathlessness, preventive anti-malarial treatment, family planning and HIV/AIDS.

The assessment of birth preparedness counseling involved observation of whether the client was counseled on planning for transport, setting aside emergency funds, preparing supplies to take to hospital and those to have at home and the advantages of giving birth at a health facility. We took note of the kind of treatment offered in which we focused on tetanus toxoid, fansidar, iron or folic acid. Finally counseling about the treatment offered was observed. Here we noted the client’s inquiries about the treatment offered and the providers’ responses and explanations given on how to take the treatment and its importance. To assess the client’s level of satisfaction with the antenatal care offered, the clients were interviewed to rate their level of satisfaction on a three level likert scale of unsatisfactory, fair and satisfactory service. The variables rated to determine the satisfaction of clients included; satisfaction with the waiting time, examination room privacy, counseling room privacy, time with health worker, information given by health worker, availability of medicine and satisfaction with the cost of services.

### Data Analysis

Data analysis was done in stata version 10, the details of the analysis process for each of the research questions are explained below

### Capacity available to deliver ANC services

To establish the human resource capacity available to deliver ANC services, staffing levels at the health facilities were compared to the ministry of health recommended staffing norms to determine existing gaps. The existing gaps were computed by subtracting the actual staff numbers for each of the cadres from the recommended staffing numbers and the percentage of the gap obtained.

To assess the availability of infection control facilities, physical infrastructure, diagnostic equipment and essential drugs, indices for each of them were generated. To generate an index, each variable used to assess the five different components of capacity were scored and then the sum of the scores were obtained. For example the infection control facilities index had eight variables in which each was given a score of 1 if present and 0 if absent. The infection control facilities index therefore had values ranging from (0-8). A similar approach was used for the generation of indices for physical infrastructure, diagnostic equipment and essential drugs. The physical infrastructure index had values ranging from (0-4), diagnostic equipment (0-5) and availability of drugs (0-4) depending on the number of variables assessed in each of them as described under the data collection section above.

### Process quality of the ANC service provision

To determine the quality of ANC service provision, indices for history taking, clients examination, tests performed, counseling for risk factors, birth preparedness, treatment provided and explanation about the treatment given were computed. The variables earlier described under each of these categories were first scored, such that a variable was given a score of one if undertaken and zero if not undertaken, and then the scores were summed under each of the broad categories described above to generate an index. History taking had index values ranging from (0-11), examination of clients (0-8), tests performed (0-6) counseling for risk factors (0-12), birth preparedness (0-6), treatment provided (0-3) and explanation about the treatment given (0-10).

To allow comparison between the health facilities, we categorized the indices of history taking, examination of clients, counseling for risk factors, and explanation about the treatment given into three categories, poor, fair and good. The quality of the ANC consultation process was graded as poor if its index value was between zero and three, fair if the value was between four and six and good if the value was above seven. While for tests performed, treatment provided and birth preparedness, the indices generated were not categorized and the results are presented in tables, graphs and text respectively.

### Patient satisfaction with ANC services

Frequency of the clients who rated the service as unsatisfactory, fair and satisfactory were obtained, standard descriptive statistics generated and the results presented in tables and graphs.

### Ethical considerations

Ethical clearance to carry out the study was obtained from the Higher Degree Research and Ethics Committee for Makerere University School of Public Health and the Uganda National Council of Science and Technology. Permission to carry out the study was also obtained at the district level and at the different local council authorities and health facilities. Informed consent was obtained from all the respondents and strict confidentiality was observed at all levels of the study process.

## Results

### Demographic characteristics of study participants

The majority of the respondents were below the age of 30 years (over 73%). Over 92% of the ANC clients were married and the majority of them were Protestants (41%). 85% of the clients had ever attended school but the majority (73%) only had primary school level of education.

### Capacity available to deliver ANC services in eastern Uganda

Table 1 shows staffing levels for maternal health services and capacity to deliver ANC services in Eastern Uganda. It shows the expected number of health workers according to the health sector strategic plan II (HSSP) of the ministry of health compared to the actual numbers in the two districts. Staffing levels were assessed for thirteen health centre IIIs and two health centre IVs.

**Table 1:** Staffing levels for maternal health services and capacity to deliver ANC services in Eastern Uganda

Staffing levels for maternal health services										
Carders	Hear Centre IIIs (13)				Health center Ivs (2)			
	Standard	Actual	Gap	%Gap	Standard	Actual	Gap	%Gap		
**M.O**	0	0	0	0.0	2	0	2	100.0		
**N.O**	13	4	9	69.2	2	2	0	0.0		
**C.O**	13	10	3	23.1	4	4	0	0.0		
**Midwifes**	26	16	10	38.5	6	4	2	33.3		
**C.A**	13	7	6	46.2	4	1	2	50.0		
**E.N**	26	9	17	65.4	6	2	4	66.7		
**N.A**	39	28	11	28.2	10	4	6	60.6		
**Total**	130	74	56		34	17	23			
**Overall% Gap**	43.1				47.1			
**Capacity to deliver ANC services**									
**Capacity variables**	**Indices n=(15)**									
	0	1	2	3	4	5	6	7	8	**Total facilities**
**Infection control facilities**		1	0	1	0	4	7	1	1	15
**Physical infrastructure**	1	1	0	2	11					15
**Diagnostic Equipment**	0	1	1	1	3	9				15
**Essential drugs**			6	6	3					15

The results indicate that there was an overall health workers’ gap of 43% in the health centre IIIs and 47% in the health centre IVs. The carders with the largest gaps in the health centre IIIs were found to be nursing officers (N.O) (69.3%), enrolled nurses (E.N) (65.4%), comprehensive nurses (C.N) (46.2%) and midwives (38.5%). While in the health centre IVs, it was among the medical officers (M.O) and nurses respectively (Table 1). Table 2 shows the capacity to deliver ANC services measured using different indices. The assesment shows that eleven out of the fifteen health facilities scored the maximum value of four for physical infrastucture availability, implying good availability of infrastructure. Similary diagnostic equipment availability was good with nine of the facilities scoring the maximum of five. However drugs availability had twelve of the health facilities with an index value of either two or three implying poor availability of drugs. Availability of infection control facilities was fair with the majority (11/15) of the facilities scoring a mid way index value of either five or six.

**Table 2:** Quality of the ANC consultation process

Consultation process variables	Number of clients	% Of clients
Client History taking		
**Poor**	104	34.8
**Fair**	138	46.2
**Good**	57	19.0
**Total**	299	100
**Client examination**		
**Poor**	25	8.4
**Fair**	241	80.6
**Good**	33	11.0
**Total**	299	100
**Counseling for risk factors**		
**Poor**	176	58.9
**Fair**	94	31.4
**Good**	29	9.7
**Total**	299	100
**Explanation about treatment offered**		
**Poor**	94	34.4
**Fair**	155	56.8
**Good**	24	8.8
**Total**	299	100

**Figure 1:**
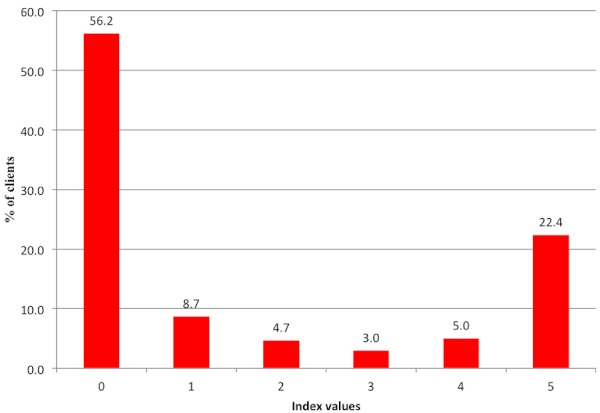
Percentage of clients observed to have undergone counseling on birth preparedness during the ANC consultation process.

**Figure 2:**
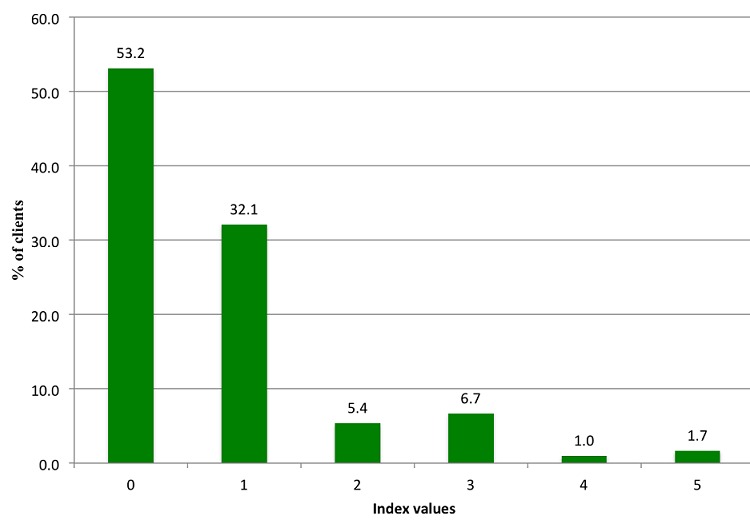
Percentage of clients observed to have had essential tests performed on them during the ANC consultation process.

Table 2 shows the percentages of clients whose consultation process was categorized as poor, fair or good for the different variables under observation. Client examination was generally well done for the majority (80%) of the clients, while counselling for risk factors was observed to have been poorly done for over 58% of the ANC clients (see table 2). Figure 1 and Figure 2 show the index values generated for counseling on birth preparedness and tests performed in the consulation process of ANC. For clients counselling on birth prepardness, over 50% (168/299) were observed to have had poor counselling. Similarly, 53% (159/299) of the clients did not have essential tests carried out on them. Nonetheless, at least 22% of the clients were observed to have undergone good counselling for birth preparedness. For the essential drugs provided, 53% (159/299) of the clients received tetanus toxoid vaccination. However the majority of the ANC clients were not offered either fansidar (62%-185/299) for intermittent treatment of malaria or folic acid (72%-215/299) as an iron supplement.

### Patient satisfaction with ANC services offered in Eastern Uganda

Table 3 shows the percentage of clients satisfied with the overall ANC services in eastern Uganda. Most of the respondents (74.6%) rated the overall ANC service as satisfactory. The variables with the most satisfied percentage of ANC clients were provider’s attitude (87.6%) and the examination room privacy (83.5%). However availability of medicines (32.3%) and waiting time (25.1%) had the highest percentages of unsatisfied clients.

**Table 3:** Percentage of ANC clients satisfied with various ANC service variables

Satisfaction Variables	Dissatisfactory	Fairly satisfactory	Satisfactory	Don’t know	Total number of clients
**Satisfaction with:**					
**Waiting time**	73(25.1%)	84(28.8%)	134(46.0%)	0	291
**Cleanliness**	12(4.1%)	75(25.8%)	204(70.1%)	0	291
**Examination room privacy**	12(4.1%)	36(12.4%)	243(83.5%)	0	291
**Consultation room privacy**	43(14.8%)	50(17.2%)	198(68.0%)	0	291
**Time given by health worker**	18(6.2%)	44(15.1%)	229(78.7%)	0	291
**Explanation given by health worker**	31(10.7%)	45(15.5%)	215(73.9%)	0	291
**Availability of medicines**	94(32.3%)	73(25.1%)	122(41.9%)	2(0.7%)	291
**Cost of service**	8(2.7%)	37(12.7%)	229(78.7%)	17(5.8%)	291
**Providers attitude**	7(2.4%)	29(10.0%)	255(87.6%)	0	291
**Overall satisfaction**	55(6.5%)	55(18.9%)	217(74.6%)	0	291
**Levels of satisfaction n= (291)**					

## Discussion

### Capacity to deliver ANC services in Eastern Uganda

The results of this study indicate that there is an overall health worker shortage of over forty percent with nursing officers having the greatest gap. Medical doctors and midwives are equally in limited supply; this is typical of the situation of health workers in Sub Saharan Africa where you find a ratio of about 15 physicians serving 100,000 people[21]. This situation is aggravated by the high rate of brain drain and the poor working conditions in low income countries like Uganda [22]. Locally the concept of brain drain is depicted in the difficulty of especially rural districts to attract health workers, eastern Uganda is one of such regions with the worst poverty indices in the country, making the region more unlikely to attract the much needed health workers [16]. The high shortage of health workers hinders the capacity of health systems to deliver the required services to its clients[23].

According to a report by Population Action International(PAI), infections account for over 15% of maternal deaths[24]. The study findings indicate that eleven of the fifteen health facilities assessed had fair infection control facilities. Good infection control is key in protecting the mother, child and health workers from hospital acquired infections[25]. The availability and proper use of infection control facliities is therefore a critical aspect of maternal health improvement that needs to be strengthened accordingly. This study also found out that availabilty of physical infrastructure and diagnostic equipment is good in the health facilities visited. This is an indication of steps taken in the right direction by government and development partners. There is however a need to ensure that there are sound maintenance and replacement polices both at national and district levels.

The study however revealed that the availability of essential drugs is generally poor, Fansidar, iron; tetanus toxoid and mebendazole are essential in the prevention of malaria, anemia, tetanus and intestinal worm infections respectively. Stock outs are a common occurrence in especially Ugandan public health facilities; this in turn frustrates efforts to improve quality of antenatal care when mothers cannot access the much needed medicines [12]. Other studies done in SSA have also reported drug shortages as a common problem [26–28].

### Process quality of ANC services in Eastern Uganda

The quality of an ANC process is critical in ensuring that the intended benefits are fully achieved otherwise the whole process loses credibility [4–7]. Tests performed, client’s birth preparedness and counseling for risk factors were the worst performed ANC processes according to this study. These findings are consistent with findings from similar studies in East Africa [17, 29]. Likewise, the Uganda demographic and health survey of 2006 reported that the provision of the full package of ANC is inadequate, for example only 35% of the clients received information on risk factors and how to recognize danger signs during pregnancy, while blood samples were taken from only 28% [12]. Nonetheless, client examination in this study was fairly done for most of the clients; this could be explained by the good availability of infrastructure and diagnostic equipment (table 2).

Although this study found the availability of diagnostic equipment to be good, it did not translate into tests being performed on clients. The probable reasons for this discrepancy could be a lack of laboratory supplies such as reagents or the mere lack of initiative to carry out these tests by the health workers and mothers as found by related studies in Kenya and Tanzania [28, 30].

### Patient satisfaction with ANC services offered in eastern Uganda

The majority of the clients interviewed rated the ANC services as satisfactory. Satisfaction with health workers attitudes which has been found by other studies [31–32] to be unsatisfactory was found to have the highest percentage of satisfied clients by this study. This needs to be understood further. However, satisfaction with waiting time and availability of medicines were rated by the highest percentage of clients as dissatisfactory. This could be explained by the fact that most of the clients sought care in public facilities which are characterized by long queues and frequent medicine stock outs compared to the private for profit and private not for profit facilities [16].

## Conclusion

This study gives important baseline information that could be used in informing the intervention design and implementation of projects that seek to improve maternal health. Major gaps exist in the staffing levels, adequacy of infection control facilities, and provision of the full package of ANC especially with regard to carrying out essential tests, counseling for risk factor recognition and birth preparedness. The erratic availability of drugs and supplies coupled with the long waiting hours are also major challenges.

The district health teams should work with facility managers to ensure the availability and use of infection control facilities. Refresher training of health workers, assesment of workloads and recruitment of more health workers is recommended as a means of ensuring the provision of a quality ANC consultation processes with satisfactory history taking and counselling sessions. Lastly but not least, adequate and consistent supply of drugs and supplies should be ensured through investing more resources in procuring them and improving on the supply chain efficiency at all levels of procurement and supply.

## References

[1] Bhutta ZA, Chopra M (2010). Countdown to 2015 decade report (2000-10): taking stock of maternal, newborn, and child survival. Lancet.

[2] WHO (2010). Trends in Maternal Mortality: 1990 to 2008, Estimates developed by WHO, UNICEF, UNFPA and the World Bank.

[3] van Eijk A (2006). Use of antenatal services and delivery care among women in rural western Kenya: a community based survey. Reproductive Health.

[4] Magadi M, Madise N, Diamond I (2001). Factors associated with unfavourable birth outcomes in Kenya. Journal of Biosocial Science.

[5] WHO WHO, programme to map best reproductive health practices.

[6] Wirth M (2008). “Delivering” on the MDGs?: Equity and Maternal Health in Ghana, Ethiopia and Kenya. East African Journal of Public Health..

[7] Nikiema L (2010). Quality of Antenatal Care and Obstetrical Coverage in Rural Burkina Faso.

[8] Chakraborty N (2002). Utilisation of postnatal care in Bangladesh: evidence from a longitudinal study. Health & Social Care in the Community..

[9] Villa J (2001). WHO antenatal care randomised trial for the evaluation of a new model of routine antenatal care. Lancet..

[10] TDHS (2005). Tanzania demographic and health survey.

[11] KDHS (2010). Kenya demographic and health survey.

[12] UDHS (2006). Uganda demographic and health survey.

[13] Chowdhury A, Mushtaque R, Mahbub Amina, Sharif C A (2003). Skilled Attendance at Delivery in Bangladesh: An Ethnographic Study.Research Monograph Series vol. 22.

[14] Pallikadavath S, Foss M, Stones RW (2004). Antenatal care: provision and inequality in rural North India. Soc Sci Med.

[15] Mathole T (2004). A qualitative study of women’s perspectives of antenatal care in a rural area of Zimbabwe. Midwifery.

[16] MOH (2006). Uganda demographic and Health survey.

[17] Sarker M (2010). Quality of antenatal care in rural southern Tanzania: a reality check. BMC Research Notes.

[18] Carroli G, Rooney C, Villar. J (2001). How effective is antenatal care in preventing maternal mortality and serious morbidity? An overview of the evidence. Paediatric and Perinatal Epidemiology..

[19] MOH (2006). The national policy guidelines and service standards for sexual and reproductive health and rights.

[20] Mcdonagh M (1996). Is antenatal care effective in reducing maternal morbidity and mortality?. Health Policy and Planning..

[21] WHO (2006). The World Health Report 2006 - working together for health.

[22] NASAC (2009). Brain drain in Africa. http://www.nationalacademies.org/includes/NASACbraindrain09.pdf.

[23] Yohannes Kinfu (2009). The health worker shortage in Africa: are enough physicians and nurses being trained?. Bulletin of the World Health Organization.

[24] PAI Population Action Plan, Maternal and Newborn health, dying for life. http://populationaction.org/wp-content/uploads/2012/02/PAI_1293-MATERNAL_compressed.pdf.

[25] WHO (2002). Prevention of hospital acquired infections-a practical guide.

[26] Boller C (2003). Quality and comparison of antenatal care in public and private providers in the United Republic of Tanzania. Bull World Health Organ.

[27] Kruk M (2009). Bypassing primary care facilities for childbirth: a population-based study in rural Tanzania. Health Policy Plan.

[28] Mwaniki P, Kabiru E, Mbugua G (2002). Utilisation of antenatal and maternity services by mothers seeking child welfare services in Mbeere District, Eastern Province, Kenya. East Afr Med J.

[29] Mutiso S, Qureshi Z, Kinuthia J (2008). Birth preparedness among antenatal clients. East Afr Med J..

[30] von Both B (2006). How much time do health services spend on antenatal care? Implications for the introduction of the focused antenatal care model in Tanzania. BMC Pregnancy Childbirth.

[31] Mrisho M (2009). The use of antenatal and postnatal care: perspectives and experiences of women and health care providers in rural southern Tanzania. BMC Pregnancy and Childbirth..

[32] Nabukera SK, Witte K, Muchunguzi C (2006). Use of postpartum health services in rural Uganda: knowledge, attitudes, and barriers. J Community Health.

